# Preheating of urine improves the specificity of urinary cryptococcal antigen testing using the lateral flow assay

**DOI:** 10.1371/journal.pntd.0005304

**Published:** 2017-05-11

**Authors:** Fábio Brito-Santos, Marcela de Faria Ferreira, Luciana Trilles, Mauro de Medeiros Muniz, Valdiléa Gonçalves Veloso dos Santos, Filipe Anibal Carvalho-Costa, Wieland Meyer, Bodo Wanke, Márcia dos Santos Lazéra

**Affiliations:** 1 National Institute of Infectious Diseases Evandro Chagas (INI), FIOCRUZ, Rio de Janeiro, Brazil; 2 Oswaldo Cruz Institute, FIOCRUZ, Rio de Janeiro, Brazil; 3 Centre for Infectious Diseases and Microbiology, Sydney Medical School-Westmead Hospital, Marie Bashir Institute for Infectious Diseases and Biosecurity, The University of Sydney, Westmead Institute for Medical Research, Sydney, New South Wales, Australia; University of Tennessee, UNITED STATES

## Overview

A delay in laboratory diagnosis is related to sequelae and death. Thus, early diagnosis is the key to decrease the high lethality rate due to cryptococcosis. The cryptococcal antigen lateral flow assay (CrAg LFA) Immy test is standardized for a fast screening point-of-care test for cryptococcosis, detecting cryptococcal antigen in serum and cerebrospinal fluid (CSF). Urine screening would be ideal as a noninvasive approach, but previous studies have shown that fresh urine present false positive results. For this reason, we introduced just one simple physical procedure (i.e, heating of fresh urine prior to CrAg LFA Immy testing) to increase the specificity without compromising test sensitivity.

## Introduction

Cryptococcosis by *Cryptococcus neoformans* is a major opportunistic infection in HIV patients, responsible for a high lethality (13%–44%), mainly in resource-limited countries. In sub-Saharan Africa, over 500,000 deaths due to cryptococcal meningitis are estimated to occur each year [1]. In Brazil, besides cryptococcosis associated with HIV, another public health problem is the endemic occurrence of cryptococcal meningitis by *C*. *gattii* in north and northeast regions with a lethality rate of 35%–40% [2]. A delay in laboratory diagnosis is related to sequelae and death. Thus, early diagnosis is the key to decrease the high lethality rate due to cryptococcosis.

Previous studies that used a cryptococcal antigen lateral flow assay (CrAg LFA) Immy in serum, plasma, finger stick whole blood, and cerebrospinal fluid (CSF) demonstrated its high sensitivity and specificity for screening cryptococcosis [3,4]. Recently, a review on diagnostic accuracy of the CrAg LFA showed a sensitivity of 85.0% (95% CI, 78.7%–90.1%) in urine. However, the specificity was not estimated [3]. A urine sample is easier to obtain than blood or CSF, becoming a promising methodology for early diagnosis of cryptococcosis, especially in developing countries [3,4]. Considering that the *Cryptococcus* target molecule glucuronoxylomannan (GXM) is thermostable, we included a heating step before the CrAg LFA Immy test to overcome the false positive results in urine, as shown in previous studies.

## Methods

A prospective cohort study performed from April 2014 to April 2015 included all HIV-positive patients over 18 years of age with CD4^+^ T cell counts ≤200 cells/mm^3^ admitted at the Evandro Chagas National Institute of Infectious Diseases (INI), FIOCRUZ, Rio de Janeiro, Brazil. Healthy individuals were included as a negative control and patients presenting proven cryptococcosis as a positive control. All patients were invited to participate in this study and provided written informed consent. The study was conducted with the approval of the INI Ethics Committee (CAAE: 3248151400005262).

Cryptococcal antigen testing was performed in blood serum and urine from each volunteer using the CrAg LFA Immy test (Immuno-Mycologics, Norman, Oklahoma, USA), following the manufacturer´s instructions. The CrAg LFA in serum samples was considered as the gold standard. Each fresh urine sample was tested under two conditions: unheated (untreated) and heated (treated) by five minutes incubation at 100°C. Clinical specimens such as blood, CSF, and urine were subsequently cultivated to investigate cryptococcal infection. Sensitivity, specificity, positive predictive values (PPVs), negative predictive values (NPVs), and Kappa statistic were determined at 95% CI by SPSS version 18.0.2 (IBM).

## Results

The study was performed on a prospective cohort of 77 volunteers: 53 HIV-positive (CD4^+^ T cell <200) patients, 18 healthy individuals (negative controls), and 6 HIV-positive patients with active proven cryptococcosis (positive controls).

Twenty-four out of 53 HIV-positive volunteers had a CrAg LFA–positive profile (42.3%) when untreated fresh urine was tested. When heated, only eight samples were positive (15%), presenting 100% of agreement with the positive results obtained from serum samples submitted to CrAg LFA Immy assay. Out of those eight positive patients, five had proven cryptococcosis (positive culture for *C*. *neoformans* in CSF and/or blood culture) and three patients had cryptococcal antigenemia (negative for *Cryptococcus* in blood and CSF cultures). The untreated fresh urine has shown 16 false positive results (30.2%). After treatment, those urine samples were negative, as confirmed by negative results in the serum.

The positive control group was positive in serum samples, in untreated and treated urine. Furthermore, *C*. *neoformans* was isolated from the clinical specimens in all cases in this group.

Thirteen out of 18 samples from the negative control group had the untreated urine positive (72%), representing false positive results, because after heating those urine samples were negative, resulting in 100% agreement with the CrAg LFA results obtained in serum.

Comparing to the serum as the gold standard, the CrAg LFA using untreated urine had a sensitivity of 100% (14/14), a specificity of 41% (26/63), a PPV of 27% (14 /51), and an NPV of 100% (26/26), whereas the comparison of serum and treated urine had a sensitivity, specificity, NPV, and PPV of 100%. Kappa coefficients (k) demonstrated a fair agreement between the methodologies by using untreated urine and serum (k = 0.204). However, perfect agreement between treated urine and serum (k = 1) was observed (**Table 1**).

**Table 1:** Summary of the diagnostic performance of cryptococcal antigen lateral flow assay on urine samples using serum as the reference standard.

Population (*N* = 77)	Sensitivity	Specificity	PPV	NPV	Kappa
Untreated urine	100% (14/14)	**41% (26/63)**	**27% (14/51)**	100% (26/26)	0.204 (*p* = 0.02)
(95% CI)	(99%–100%)	**(30%–52%)**	**(17%–37%)**	(99%–100%)	(0.068–0.339)
Treated urine	100% (14/14)	100% (63/63)	100% (14/14)	100% (63/63)	1.0 (*p* < 0.001)
(95% CI)	(99%–100%)	(99%–100%)	(99%–100%)	(99–100%)	(0.755–1.0)

Data presented are the percentage, numerator/denominator, and 95% confidence interval.

Kappa, analysis of agreement by Kappa index; NPV, negative predictive value; PPV, positive predictive value.

## Discussion

Early diagnosis and treatment is an important strategy for preventing clinical disease and reducing the high lethality rate in HIV patients with detectable CrAg. Urine specimens are easier to collect than other samples, providing a convenient way to screen suspected patients, especially in remote areas with resource-limited settings without laboratory facilities.

As shown herein and in a study by Tenforde et al. (2015) [5], fresh urine samples should not be used in point-of-care settings for screening of cryptococcosis. The significant number of false positives in fresh urine can induce a false diagnosis of cryptococcosis and the misuse of antifungal drugs [6]. Early studies on the CrAg LFA test showed high sensitivity and low specificity in urine. [7,8,9]. In other studies, the diluent was changed or the urine was frozen in order to improve specificity but the results didn’t reach the gold standard pattern (serum, CSF) [8,9]. The present study calls attention on the unexpected high number of false positives in fresh, untreated urine samples from healthy individuals included as a negative control and also from HIV patients. Considering a possible cross-reaction with some macromolecules, which are part of the usual profile of the urine, and taking into account the presence of thermosensitive molecules, we propose a fast and reliable method of inactivation of those molecules (i.e., heating fresh urine at 100°C for five minutes before the CrAg LFA), which has no effect on thermostable cryptococcal GXM antigen—the major component of the capsule. Heating of urine prior testing dramatically improved the test’s specificity without compromising the test’s sensitivity. The CrAg LFA in heated urine of HIV patients identified not only five new cases of cryptococcosis but also three cases of cryptococcal antigenemia. Further studies on CrAg test are necessary to understand which molecule or molecules could be acting as interference, producing cross reactivity in urine.

In conclusion, this crucial step has dramatically increased the specificity without compromising test sensitivity, with two additional advantages: no need of enzymatic treatment or sample dilution (box 1).

**Box 1. Advantages and disadvantages of heating urine prior CrAg LFA tests**

Advantages

- High specificity and sensibility as the gold standard technique (with serum).
- Easy implement without need for phlebotomy or finger stick supplies.
- Screening early cryptococcal infection at routine laboratory as well as in field work.

Disadvantages

- One more step is included in the methodology: the boiling.
- Five more minutes are added to the methodology.
- Cannot be a point-of-care test.
